# Estimation of Radiofrequency Power Leakage from Microwave Ovens for Dosimetric Assessment at Nonionizing Radiation Exposure Levels

**DOI:** 10.1155/2015/603260

**Published:** 2015-02-01

**Authors:** Peio Lopez-Iturri, Silvia de Miguel-Bilbao, Erik Aguirre, Leire Azpilicueta, Francisco Falcone, Victoria Ramos

**Affiliations:** ^1^Electrical and Electronic Engineering Department, Universidad Pública de Navarra, Pamplona, 31006 Navarra, Spain; ^2^Telemedicine and eHealth Research Unit, Health Institute Carlos III, 28029 Madrid, Spain

## Abstract

The electromagnetic field leakage levels of nonionizing radiation from a microwave oven have been estimated within a complex indoor scenario. By employing a hybrid simulation technique, based on coupling full wave simulation with an in-house developed deterministic 3D ray launching code, estimations of the observed electric field values can be obtained for the complete indoor scenario. The microwave oven can be modeled as a time- and frequency-dependent radiating source, in which leakage, basically from the microwave oven door, is propagated along the complete indoor scenario interacting with all of the elements present in it. This method can be of aid in order to assess the impact of such devices on expected exposure levels, allowing adequate minimization strategies such as optimal location to be applied.

## 1. Introduction

A new age based on progress, increasing production, consumer consumption, and the increasing exploitation of nature is leading to a never-ending addition to human knowledge and wellbeing. But during the 1960s the first doubts began to emerge, coinciding with the birth of the environmental movement in America. A thought began to spread widely in the public awareness about the unforeseen hazards caused by products of our modern technological world that had an adverse impact on quality of life [1].

The risk management changed due to a reflexive modernization. Previously, unintended risks of the industrial sector remained largely hidden and marginalized with a public consensus that all social, environmental, and political risks could be solved simply by the existing scientific and technological expertise. However, when the environmental risks created by the industrial sector of society moved to the center of public attention in the 1960s–1970s, the industrial sector took a different viewpoint about the risks they needed to address and manage [2]. Reflexive modernization was characterized by an increasing public awareness and concern over the negative consequences of industrial and technological development [3]. The uses of radio frequency (RF) and microwave energy in communications and industrial and medical applications were a burgeoning economic sector. A common theme in the industry responses was a stated belief that the standard assured that all RF emitting technologies were safe as long as exposures were kept below the recommended limits.

National governments in several countries and health authorities have been urged to adopt measures to prevent, or to minimize, risk associated with electromagnetic field (EMF) exposure. Standards on protection against possible health effects of EMF have been developed and updated by various international and national bodies for several decades. Over the years, such standards have evolved from simple recommendations on exposure limits in a limited frequency range to a comprehensive and complex system of protection, covering a large part of the spectrum of nonoptical EMF (in general, from 0 Hz to 300 GHz) [4].

There are many partners contributing to this process including World Health Organization (WHO), the International Commission on Nonionizing Radiation Protection (ICNIRP), and national agencies and advisory bodies. In response to that interest, in 1998 the ICNIRP, a panel of experts commissioned by WHO, published a group of guidelines aimed at limiting human exposure to nonionizing electromagnetic fields with potentially harmful, short-term health effects. A wide consensus exists on those guidelines that have formed the basis for national regulations in several countries.

The ICNIRP guidelines, as well as other international standards, are based on a two-level structure. Basic restrictions are defined in terms of “dosimetric quantities”—in particular current density for low-frequency electric and magnetic fields [5, 6] and specific absorption rate (SAR) and power density for high-frequency (10 GHz) electromagnetic fields—that are directly related to biological effects.

The ICNIRP guidelines were the basis of the current regulations not only in European countries, but also in the rest of the world. Electromagnetic field standards in the Western European countries are based on well-established acute biological effects that could be considered as signaling of potentially adverse health effects, with the specific absorption rate being the basic restriction of exposure to RF fields. On the other hand, Eastern European (EE) standards are designed to protect from potential nonthermal effects that might be caused by chronic exposure to very low intensities. Thus, EMF standards in EE countries differ considerably from those which are proposed by the ICNIRP and the IEEE Standard C95.1. Differences in the exposure limit are over two orders of magnitude between EE and Western European countries. In general, the EE standards are set at considerably lower level. In fact, frequently the EE standards allow considerably higher EMF levels at certain frequencies although for shorter period of time than what is permitted for workers in Western standards [4]. The Western standards are primarily based on the concept of a thermal mechanism and protect only against “thermal” effects. In EE countries, in contrast to western approach, not only the thermal effects but also the numerous reactions (which do not necessarily lead to adverse health effects) of the organism without a temperature increase following prolonged exposure [7] are considered. Overall, existing differences in EE and Western safety standards could be attributed to dissimilarities in all stages of the standards setting process: from methodology employed in experimental studies to different philosophies for standards development and the scientific data used as basis for standards including risk perception and risk acceptance.

In USA internationally recognized standards have been developed by other bodies, in particular the Institute of Electrical and Electronic Engineers (IEEE), and the Federal Communications Commission (FCC) enforces limits for both occupational exposures (in the workplace) and public exposures. In spite of few differences of some importance, such as the one- or two-tier (workers versus general public) structure, or the classification of the environments rather than of the exposed personnel, these standards show close similarities and are based on the same approach and rationale as ICNIRP guidelines. The exposure limits can differ by factors of 10 or more, depending on the frequency range and the exposed public (workers, general public). Among the factors that contribute to the differences between countries are the selection and interpretation of data, the reasons for which standards have been set, and the sociopolitical context which may influence the level of application of precautionary principle.

About the current regulation in the rest of the world, the most commonly used safety standards at the present time are the ICNIRP and ANSI/IEEE C95.1 (ICNIRP 1998; IEEE 1999) [8, 9]. In North America, the contents of the Canadian regulations are a slightly modified version of the IEEE Standard C95.1. About South America, the Pan American Health Organization is promoting scientific research, often in the form of epidemiologic studies, in order to propose uniform norms and standards. Some Latin American countries, including Argentina, Brazil, Chile, Colombia, Costa Rica, Ecuador, Mexico, Peru, and Venezuela, have already enacted incomplete or partial legislation based on recommended international standards. South Africa is the only African country that is provided with known rules about the exposure to EMF. Protection against EMF is provided by a voluntary compliance with ICNIRP guidelines, for both general public and workers. In Asia, most of the countries have not adopted known standards. In Israel, current limits are based on ICNIRP guidelines, although revisions have been carried out. For the general public only, a safety factor is added for environmental guidelines. In 2001, in Turkey a regulation of EMF exposures of general public in the frequency range 10 kHz–60 GHz has been implemented. Reference levels (electric field, magnetic field, and power density) and safety distances are provided, with no use of dosimetric quantities. Only in case of multiple frequency exposures, ICNIRP guidelines are implemented. In Japan, guidelines in the frequency range 10 kHz–300 GHz are consistent with ICNIRP guidelines, but not exactly the same. About the rest of Asian countries the following ones are provided with known specific rules: Singapore, China, South Korea, Taiwan, and Philippines. Singapore has adopted safety guidelines on EMF exposure based on the ICNIRP, of voluntary compliance for workers and general public. In China a hygienic standard was issued by the Ministry of Health, which consists of exposure limits on mandatory compliance for public and occupational environments. In the Republic of Korea the current rules are guidelines of public and occupational compliance for protection from exposure and for devices and methods to measure EMF and guidelines for measurement of SAR of public compliance. Philippines have adopted Radiation Protection Standards for Radiofrequency based on the Australian Standard. In 2002, Australian Radioprotection and Nuclear Safety Agency (ARPANSA) published the standard: Radiation Protection Standard for Maximum Exposure Levels to Radiofrequency Fields—3 kHz to 300 GHz, where limits specified are based on the ICNIRP 1998 Guidelines. The standard also includes requirements for protection of the general public and the management of risk in occupational exposure, together with additional information on measurement and assessment of compliance [10].

Despite the ubiquity of new technologies using RFs, little is known about population exposure from RF sources and even less about the relative importance of different sources. Other cautions are that microwave oven studies to date have been able to address only relatively short lag periods that almost no data are available on the consequences of exposure and that published data largely concentrate on a small number of outcomes and without going into depth on the specific issue of the microwave oven [11–15].

In this paper, the domestic microwave oven has been analyzed as a RF source in terms of nonionizing radiation dosimetry. Since the first appearance of the microwave oven in 1945 [16], developed by Raytheon Manufacturing Company, a high penetration of this appliance has taken place and today they can be found in most of the homes and buildings around the world. This technology represents an easy and fast way for heating food. Due to that, it has been exported to other areas, as the industry, where microwave ovens are used for the drying of different kinds of products [17–19] among other utilities [20].

Due to the high penetration of the microwave oven, extensive bibliography can be found. Both the electromagnetic propagation inside the cavity and outside the oven has been studied. The design of the oven has an important impact on the efficiency, as it affects the electric field distribution inside the cavity [21–24]. Avoiding the power leakage, which mainly occurs through the door, has been a key issue [25, 26], and that is why special attention has been paid to the design of the oven door [27–30]. Typically, the leakage has been treated as interference for devices like pacemakers [31] and wireless communication systems (Wifi, ZigBee, and Bluetooth), as the microwave oven operates in the ISM (industrial, scientific, and medical) frequency band of 2.4 GHz [32–34]. But there are more scenarios where the leakage of the oven has been analyzed, as restaurants, where microwave ovens are more powerful [35], or in highly confined scenarios, such as airplanes [36].

Aside from the analysis of the leakage and its possible effects on different technologies and scenarios, the modeling and the simulation of the leaked power have great relevance in order to predict its behavior, being able to identify potential problems when deploying a wireless network. In the literature theoretical, practical, and statistical methods for calculating the interference produced by the oven can be found [37–39]. This prediction of the leakage is usually performed by CAD methods and 3D electromagnetic field simulations [40, 41].

In this work a comprehensive study on dosimetry of domestic microwave ovens is presented. An analysis of the leaked electric field has been performed taking measurements in an indoor scenario and comparing them to the simulation results obtained by a complex novel method. The simulations have been done by means of CST Microwave Studio software combining with a 3D ray launching algorithm, which has been implemented in-house. In Section 2 the ray launching technique is presented, and Section 3 describes the full simulation methodology for the microwave oven. In Section 4 measurements are presented, where both a dosimeter and a spectrum analyzer results are shown. Once the prediction and measurements of the leaked electric field have been done, in Section 5, the obtained values have been compared with the ICNIRP scale in order to see if they comply with the corresponding human exposure level. For a more in-depth study of the human exposure to an operating microwave oven, SAR values on a human body have been estimated.

## 2. In-House Ray Launching Simulation Technique

Prediction of radio-propagation behavior is becoming crucial to wireless systems design. Since site measurements are costly, propagation models have been developed as a suitable, low-cost, and convenient alternative. Traditionally, empirical methods were used (such as COST-231, Walfish-Bertoni, Okumura Hata, etc.) for initial coverage estimation [42–44]. They are simple and efficient to use, but they cannot be used for different environments without modification. On the other hand, there are deterministic methods [45–51], which are founded on the resolution of Maxwell's equations, as ray launching/ray tracing (based on geometrical approximations) or full-wave simulation techniques (method of moment (MoM), finite difference time domain (FDTD) [52], etc.). The input parameters of these methods can be very detailed and accurate. Nevertheless, the drawback is the large computational overhead that may be prohibitive for some complex environments. As a midpoint, offering a reasonable commitment between precision and required computation time, there are methods based on geometrical optics, allowing radio planning calculations with strong diffractive elements [53]. The ray tracing method combined with uniform theory of diffraction (UTD) [54] is most frequently applied to radio coverage prediction [55–58]. The ray tracing models potentially represent the most accurate and versatile methods for urban and indoor, multipath propagation characterization or prediction.

**Figure 1:**
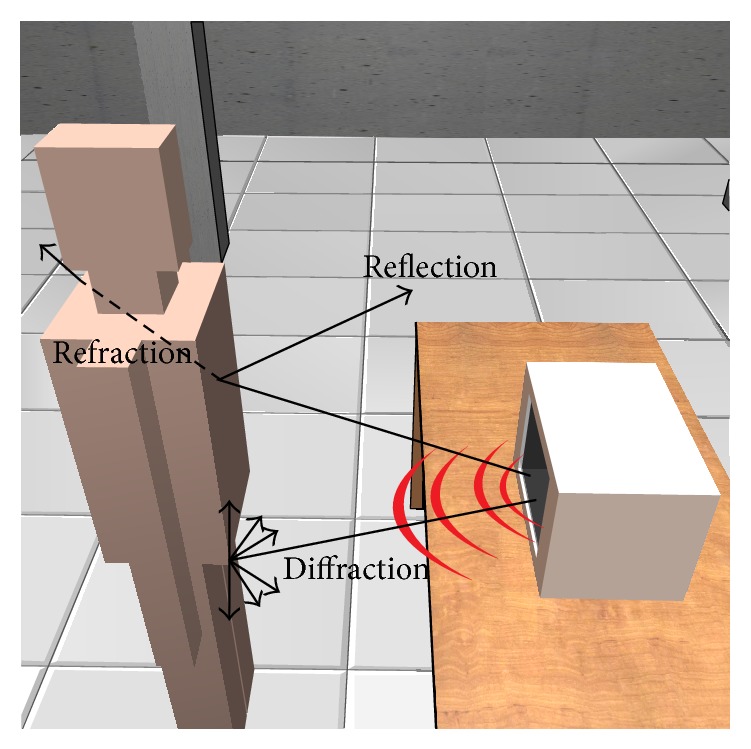
Principle of operation of the 3D ray launching method implemented in-house to perform indoor coverage analysis.

In this study, the dosimetric analysis of the propagation channel due to microwave oven leakage has been performed with the aid of a 3D ray launching algorithm, which has been implemented in-house based on Matlab programming environment. The presented simulation method has been widely validated in different kind of complex indoor scenarios [59–63]. The principle of the ray launching method is to launch a bundle of rays from the transmitter at an elevation angle and with an azimuth angle as defined in the usual coordinate system. The number of rays considered and the distance from the transmitter to the receiver location determine the available spatial resolution and, hence, the accuracy of the model. A finite sample of the possible directions of the propagation from the transmitter is chosen and a ray is launched for each such direction. When the launched ray interacts with an obstacle, reflection, transmission, and diffraction will occur, depending on the electric properties and the geometry of the impacted object, as is depicted in Figure 1.

Antenna patterns are incorporated to include the effects of antenna type. Parameters such as frequency of operation, number of multipath reflections, cuboid dimensions, or separation angle between rays are specified. The material properties for all the elements within the scenario are also taken into account, given the dielectric constant and permittivity at the frequency range of operation. All these parameter possibilities make the presented simulation method a high accurate tool for obtaining radiated power estimations within complex indoor scenarios in an acceptable computational time.

## 3. Simulation Methodology

**Figure 2:**
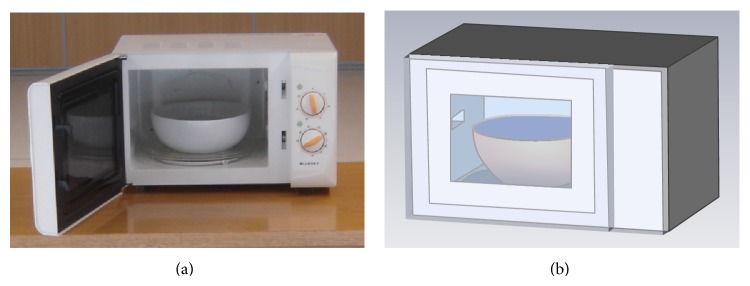
The microwave oven used for the measurements (a) and the schematic view of the created simulation model (b). Note that a porcelain bowl, filled with water, is placed in the center of the cavity for both the measurements and simulation.

In this work a common domestic microwave oven has been used, with 45.5 cm length, 27.7 cm height, and 27.5 cm depth. In order to simulate the behavior of the leaked microwave oven power in its surroundings, full wave electromagnetic results have been obtained with the aid of CST Microwave Studio. The simulation model created for that purpose can be seen in Figure 2. The real dimensions of the oven as well as the real material characteristics have been taken into account to create an accurate simulation model. Using these previous simulation results, an equivalent modeling of the oven has been done to be applied in the 3D ray launching simulation software described in Figure 1. The aim of this simulation is to fully estimate the interference created by the leaked power in a complete volume of an indoor scenario. The whole simulation procedure of the estimation of the oven leakage has been described in detail previously [64].

As can be also seen in the previously mentioned work [64], the spectrum of the leaked power exhibits a wideband nonuniform power distribution, so the radiated electric field value will be different depending on the frequency. Because of this, several simulation values have been calculated at different frequencies. As the goal of this work is to analyze the dosimetry and the level of the radiated electric field caused by a microwave oven in terms of human exposure, the simulation results shown in this paper correspond to the frequency which has the maximum power level (2.465 GHz). So the worst case is analyzed, or what is the same, the case of the highest dose is calculated.

**Figure 3:**
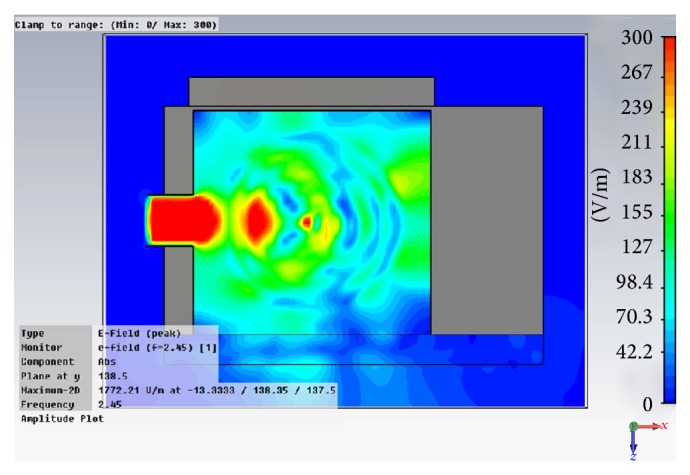
Screen capture of the upper cut taken from the CST simulation of the microwave oven. Note the leakage through the door (downside of the picture).

In Figure 3 an upper cut taken from the 3D full wave simulation is shown, where the distribution of the electric field inside the oven cavity can be seen. The amplitude of the electric field is depicted in V/m. The electric field which leaked through the front door can be seen.

**Figure 4:**
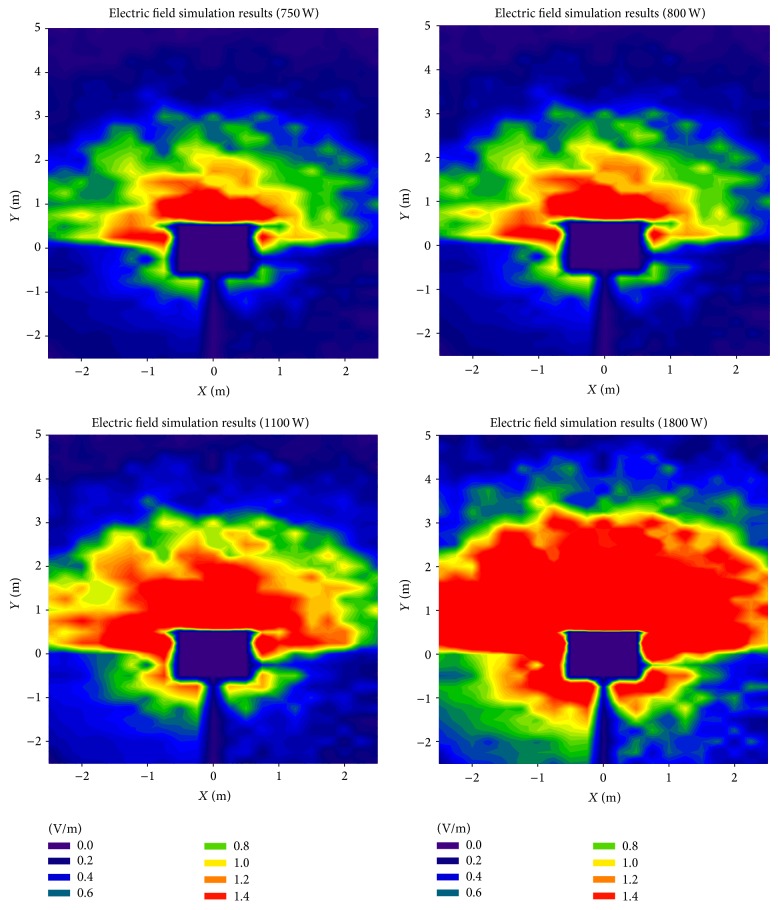
3D ray launching electric field results for a height of 0.7 m, considering different maximum output power values.

As previously stated, once the leakage around the oven has been obtained, equivalent sources have been calculated for the ray launching simulations performed afterwards. Thus, the behavior of the leaked electromagnetic power propagation can be analyzed, which will enable the estimation of the interference effect within the complete indoor scenario. It is worth noting that, in principle, the surface current distribution on the microwave oven structure exhibits a uniform distribution in terms of time dependence. Therefore, in order to gain insight on potential modifications of the microwave oven model, the main contribution is provided by considering the maximum output power available. In this sense, several cases of maximum output power values have been considered, in the range of 750 W (low power model) to 1800 W (heavy duty model), where 800 W is the model which has been measured.  Figure 4 shows the results obtained in the simulation of the complete scenario, for 4 different maximum power levels. The microwave oven is located at coordinates (0,0), with the door orientated facing the positive values of the *Y*-axis. As expected, the highest electric field values can be found in the nearest zone of the oven, particularly in front of the door, as the radiated power is mainly due to the leakage through the door. It is worth noticing that, although in lesser extent, the leaked power also affects the zone behind the oven. The radiated electric field can easily reach more than 3 meters, which is a significant distance considering indoor scenarios as home environments.

**Figure 5:**
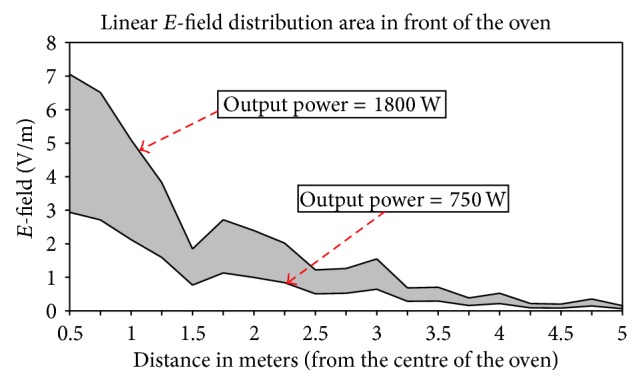
Linear* E*-field distribution in front of the oven as a function of maximum output power depending on the selected model of microwave oven.

In order to provide insight in relation to the power levels expected as a function of maximum output power, Figure 5 depicts the region, as a function of distance in which emission levels will be present, in the case in which maximum power is applied for the oven of least maximum power (i.e., 750 W) and the heavy duty model (1800 W).

## 4. Measurements Methodology

**Figure 6:**
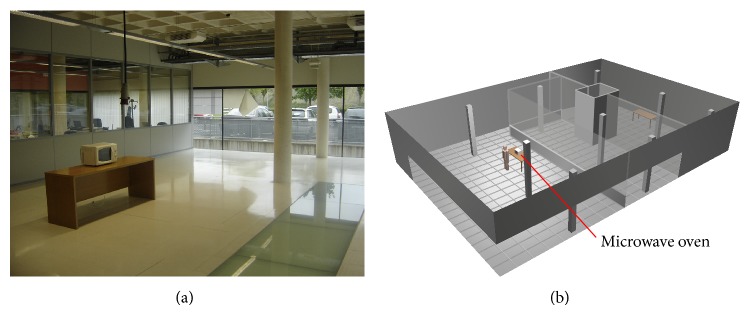
The scenario where the measurements have been taken (a) and its schematic representation for the simulation by means of 3D ray launching software (b).

In order to validate the leakage estimations for the complete scenario, measurement results have been made. The scenario in which the measurements have been taken and the simulations have been run is placed in the ground floor of the Research and Development building of the Public University of Navarre. In Figure 6 the composition of the test bed and its schematic representation for the 3D ray launching algorithm is shown, where the microwave oven has been positioned on a wooden table at height 0.7 m. The scenario has the inherent complexity of indoor scenarios due to the different elements within it, as interior columns, metallic elements, and walls made of different materials (glass, wood, concrete, and bricks).

**Figure 7:**
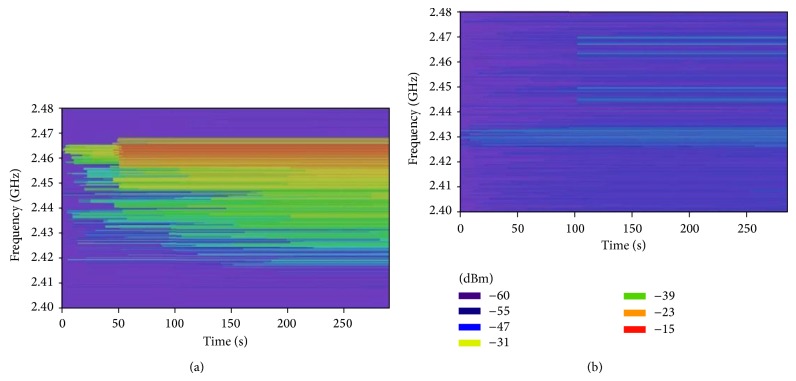
Measured spectrograms within the scenario (a) with the influence of an operating microwave oven and (b) without the oven.

In the first place, spectral measurements have been taken with the aid of an Agilent Field Fox N9912A spectrum analyzer. Specifically, two spectrograms have been obtained, the first one with the oven in operation mode and the second one without the oven. For that purpose, the receiver antenna, which has been coupled to the spectrum analyzer, has been placed separated 10 cm from the oven door. The spectrograms have been obtained for an interval of 5 minutes once the oven starts heating. The aim of these measurements is to show how the leaked power from microwave oven is very strong and how it covers almost the whole ISM band of 2.4 GHz. This can be clearly seen in Figure 7, where the left spectrogram has been taken with the oven operating at its highest power mode (800 Watts) and the right spectrogram represents the same situation but without the microwave oven.

Once the influence of an operating microwave oven has been shown, specific measurements have been taken in order to quantify the power level of the leaked electromagnetic field throughout the scenario. These measurements have been taken with the same spectrum analyzer. To carry it out, an array of measurement-points has been set. The size of this measurement zone is 5 m × 7.5 m, with the oven situated in the coordinates (0,0) shown in Figures 4 and 9. The array consists in a total of 650 measurement points taken every 0.25 meters within the measurement zone, creating an array of 21 rows × 31 columns of points. The height has been set at 0.7 m, which is the height corresponding to the center of the microwave oven. As a receiver, an omnidirectional 7 dBi antenna has been used, which provides a 360° horizontal beam width and vertical beam width of 23° (model OAN-1070 from LevelOne). The received leakage has been measured for each position and for 30 seconds, while the microwave oven was in operation. As the spectrum analyzer gives received power level in dBm, a conversion to electric field values has been required.

**Figure 8:**
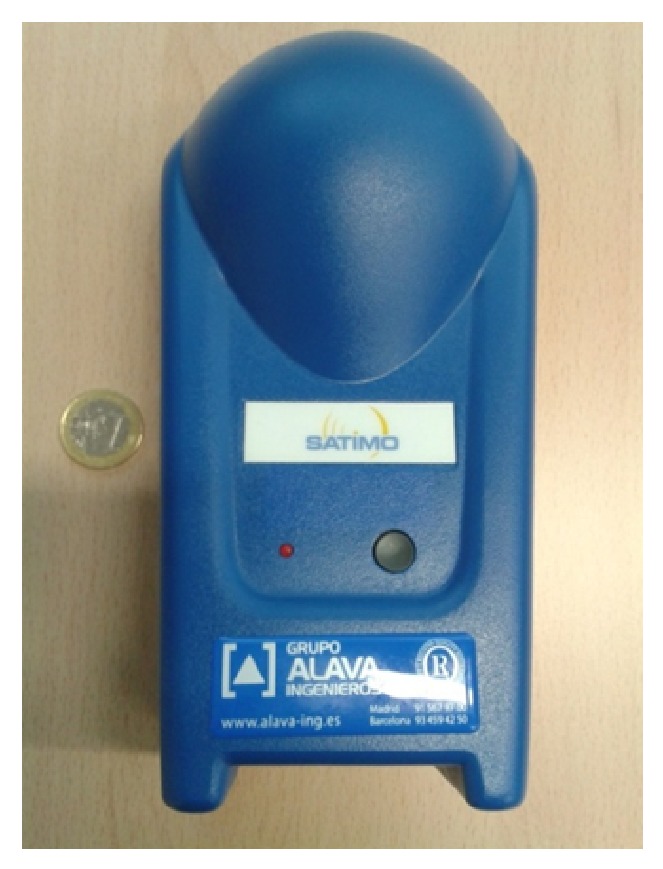
The EME Spy 121 dosimeter used to obtain electric field values of the leakage of the microwave oven.

On the other hand, the same measurements have been performed, but with an EME Spy 121 personal dosimeter (see Figure 8). This type of dosimeter is specifically designed to make selective measurements of the level of personal exposure (in terms of electric field strength in V/m). Although most regulations require gathering data at a rate of one sample per second for a measurement time of six minutes, the shortest sample rate available for the EME Spy 121 dosimeter device is one sample per 4 seconds, which has been the sample rate used for the measurements in this study. The overall measurement time for each measurement point has been set to 3 minutes instead of 6 minutes due to the big quantity of measurement points proposed for this study. Besides, the purpose of these measurements is to validate the measurement data obtained by the spectrum analyzer as well as the simulation results, proving that the presented 3D ray launching algorithm can be also used for dosimetric assessment. As can be seen in Figure 7(a), the maximum value of the microwave oven's leaked power is obtained quite early, which has been the value compared with spectrum analyzer and simulation results. Therefore, the 3 minutes set as measurement time are enough for the purpose of this part of the study.

**Figure 9:**
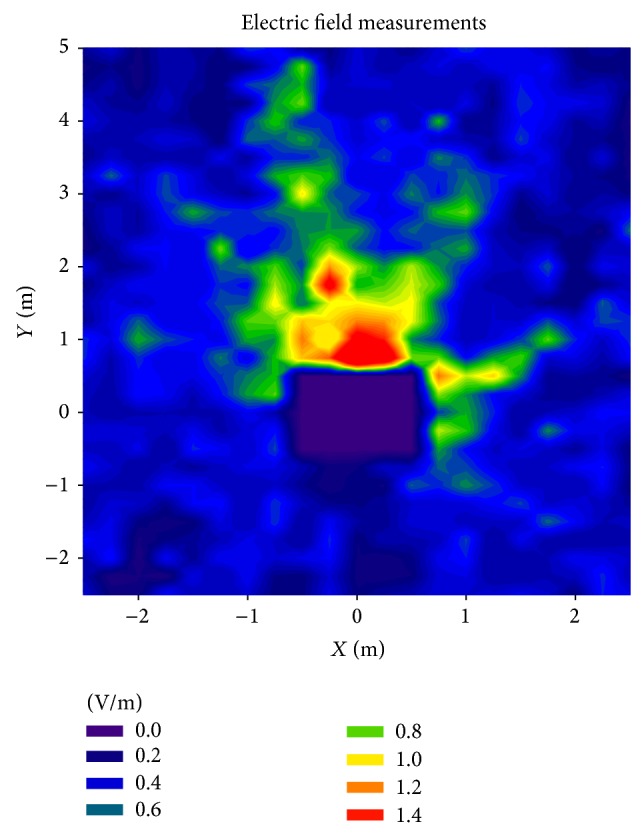
Measurement electric field values obtained with the spectrum analyzer.

The obtained measured values are shown in Figure 9. In this case, the shown data are the corresponding values obtained with the aid of the spectrum analyzer, once they have been converted to electric field values.

**Figure 10:**
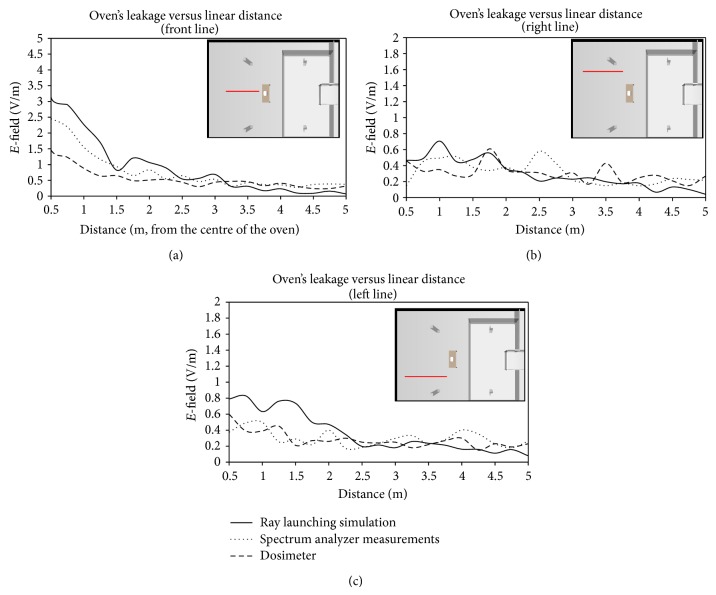
Received electric field distribution versus distance for 3 different radials ((a) front, (b) right, and (c) left). Simulation as well as measurement results from spectrum analyzer and EME Spy personal dosimeter are represented, with good agreement among them.

Although the error between the simulation (Figure 4) and the measurements (Figure 9) at first glance appears to be quite high, it is due to the low field levels and the color scale that has been needed to be used. In order to obtain a clearer representation of the comparison between simulation and measurements, three different linear power distributions with transmitter-receiver distance have been taken from the measurement/simulation zone.  Figure 10(a) represents the radiated oven leakage versus linear distance for a central line, which corresponds to a straight line just in front of the microwave oven, as can be seen in Figure 9. Figures 10(b) and 10(c) represent the comparison for left and right lines.

As can be seen in Figure 10, the electric field values obtained with the spectrum analyzer and dosimeter are heavily similar, as expected. If measurements are compared to simulation results, a high accuracy is observed. Taking into account the 650 points distributed within the scenario where the measurements have been taken, a total error mean of 0.059 V/m is obtained between simulation results and spectrum analyzer measurements. Those values and the values obtained by means of the dosimeter are also close, as shown in Figure 10. Specifically, the mean error between simulation results and dosimeter values is 0.147 V/m, higher than the error obtained by the spectrum analyzer, probably due to the loss of information derived from the method employed by the dosimeter to extract electric field samples (i.e., a sample every 4 seconds). It is worth noting how important it is to use a well-developed and detailed microwave oven for simulations in order to obtain accurate dosimetric values throughout a scenario: although the lateral lines used to show the comparison between simulation results and measurements (see Figures 10(c) and 10(b)) are symmetric with respect to the oven's cavity, the simulation results are different for both lines, as it also happens to the measurement results. The main reason for that phenomenon is that the power leaked through the oven's door is not uniformly distributed on the oven's surface, and, consequently, the radiation pattern of the whole oven is not symmetric. Besides, the scenario itself is not symmetric (see Figure 6), making the propagated multipath components different for both sides of the oven. The nonsymmetrical radiation characteristics exhibited by the microwave oven can be clearly observed in the results depicted in Figures 4 and 9.

## 5. Exposure Level Analysis

Studies on the impact of electromagnetic wave exposure on humans and different kinds of animals have led to the specification of different standards, which have been designed to set a nonionizing radiation exposure level compatible with human health. The most authoritative guidelines at international level have been developed by the ICNIRP, which has been previously commented on in this work. The ICNIRP criteria and guidelines specify limit values for occupational exposure as well as for general public exposure.

At the frequency of operation of the current work (around 2.45 GHz), the ICNIRP general public exposure limit is 61.492 V/m, and occupational exposure limit is 137 V/m. As can be seen throughout this work, the obtained electric field values are lower than 4 V/m, so far lower than the ICNIRP limit levels.

**Figure 11:**
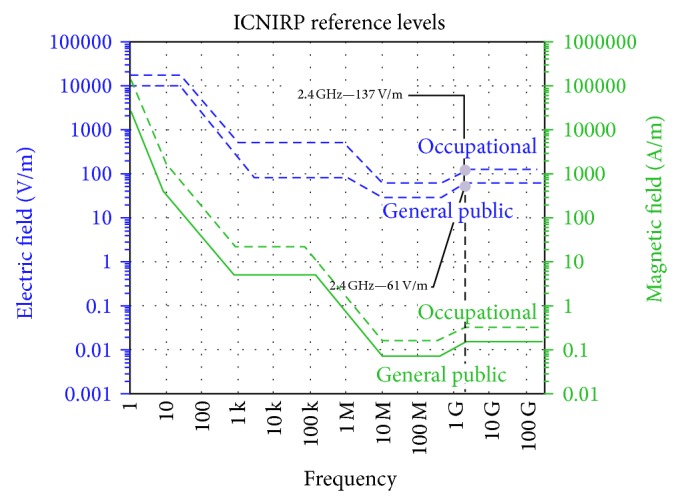
ICNIRP reference levels of the* E*-field for occupational and general public exposure. The specific level for the working frequency of 2.4 GHz has been indicated.

**Figure 12:**
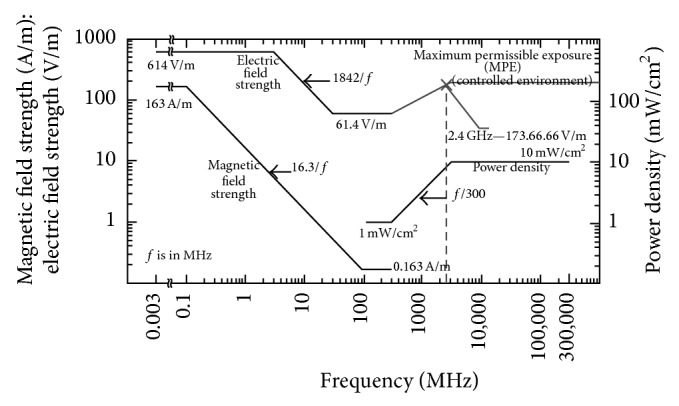
IEEE thresholds of* E*-field for controlled environments. The specific level for the working frequency of 2.4 GHz has been indicated.

**Figure 13:**
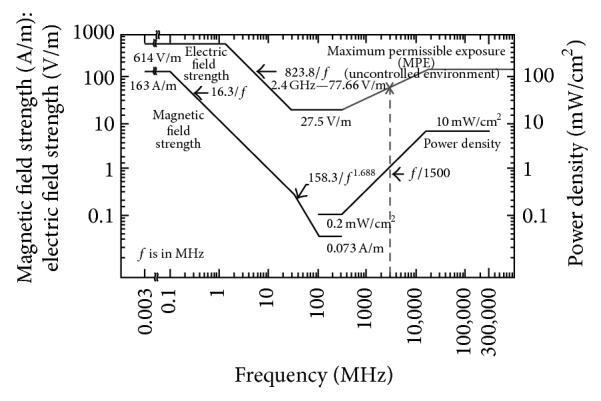
IEEE thresholds of* E*-field for uncontrolled environments. The specific level for the working frequency of 2.4 GHz has been indicated.

Figures 11, 12, and 13 show the reference levels of the* E*-field recommended by the ICNIRP and the IEEE for both controlled and uncontrolled environments.

The former Soviet Union (USSR) and the USA were the first countries to introduce standards limiting exposure to radiofrequency (RF) fields. The exposure limits in the USSR standards were always much lower than those in the USA and other countries.  Table 1 summarizes the exposure limits for the electric field that are established in the most widespread standards, ICNIRP and IEEE, in the European countries, in contrast with the established limits in Russia that are not dependent on the frequency in the range of 300–3000 MHz [65]. The exposure limits established by IEEE, ICNIRP, and the Russian Standard are indicated for the 2.4 GHz frequency band.

**Figure 14:**
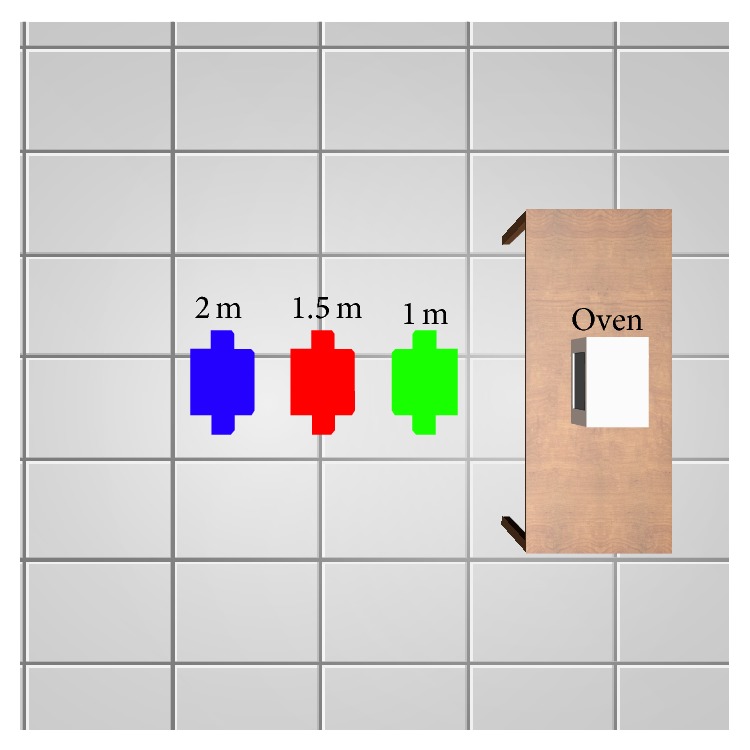
Three positions for calculating the SAR, 1 m (green), 1.5 m (red), and 2 m (blue) from the microwave oven.

In order to get the specific absorption rate (SAR) for human body, an in-house developed human body model has been used and situated in three places of the scenario doing three simulations. This human body model has been tested in several works [61, 62], giving a good accuracy in the simulation results. As seen in Figure 14 the human body model has been planted in a distance of 1 m, 1.5 m, and 2 m from the microwave oven.

The SAR value is obtained using the following expression:

(1) $$\begin{array}{c} \text{SAR}=\frac{\sigma }{\rho }{\left|\vec{E}\right|}^{2}, \end{array}$$

where conductivity (*σ*), tissue density (*ρ*), and electric field are considered. This SAR calculation method has been extracted from [66] and is implemented in some papers [67, 68]. The human body model parameters used in this study, as the age (35–39 years old), the height (1.80 m), and the fat percentage (26.9%), have been chosen taking into account previously published works, as well as conductivity of the skin (10.18 S/m) [69] and the human body density (1043 Kg/m^3^) [70].

**Figure 15:**
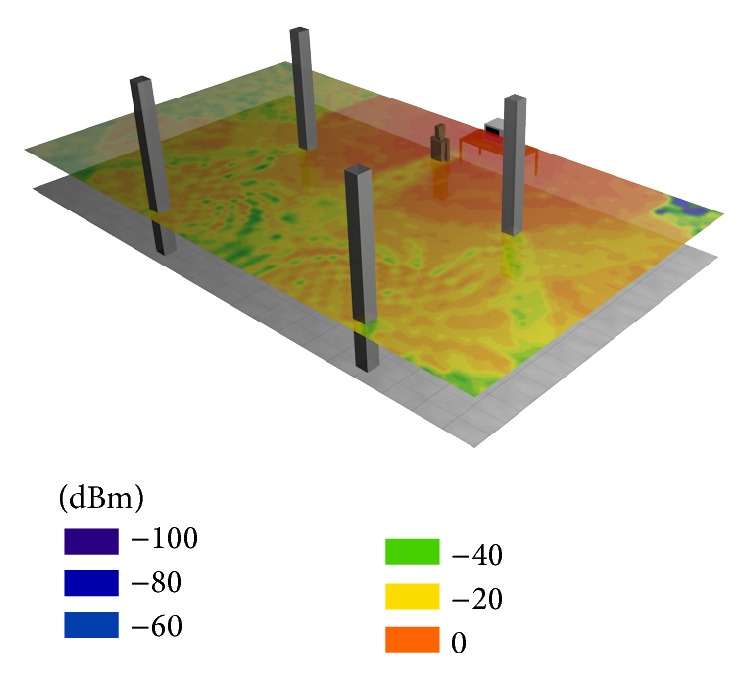
Power distribution for the part of the scenario where human body is placed when it is at 1.5 m distance from the microwave.

In Figure 15 the power distribution for this scenario is depicted. For this picture the scenario has been simplified because the interesting zone is in front of the microwave oven where the human body is located. The influence of the presence of the human body model in the overall radiated power distribution within the complete scenario can be seen, as lower power levels appear in the regions behind the human body model, depicted in Figure 15.

**Figure 16:**
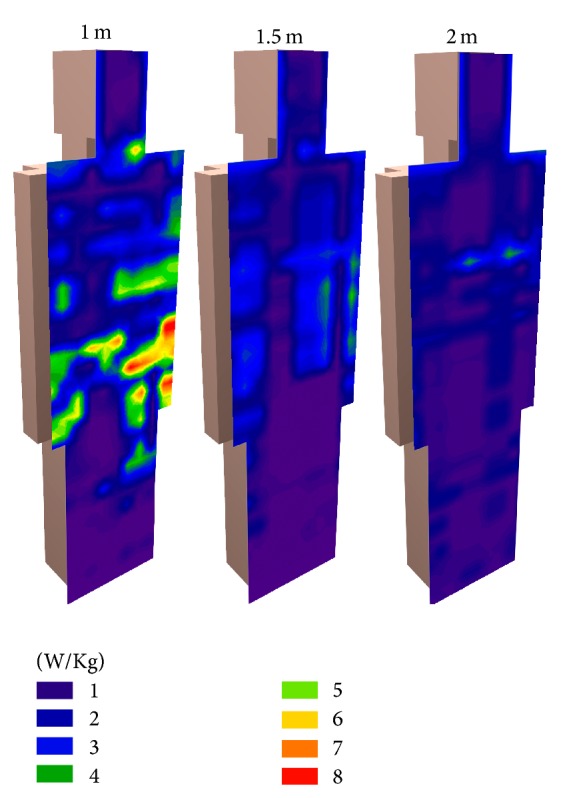
Estimation of SAR values for human body model situated in 1 m, 1.5 m, and 2 m.

In Figure 16 SAR results in body for three different simulations are depicted. As expected, higher absorption has been produced when the human body is closer to the energy source.

To protect people from EMF overexposure, the ICNIRP and the IEEE have defined limits that have been established in the great majority of countries in the world. The restrictions in these guidelines were based on scientific data alone; currently available knowledge, however, indicates that these restrictions provide an adequate level of protection from exposure to time-varying EMF. Two classes of guidance are presented in ICNIRP, which is the most widespread and used standard [8].

- *Basic Restrictions*. Restrictions are based directly on established health effects. Some of the physical quantities used to specify these restrictions are the SAR, expressed in watts per kilogram, and the power density (S), in W/m^2^.
- *Reference Levels*. These levels are provided for practical exposure assessment purposes to determine whether the basic restrictions are likely to be exceeded. Some reference levels are derived from relevant basic restrictions using measurement and/or computational techniques and some address perception and adverse indirect effects of exposure to EMF. One of the derived quantities is the electric field strength.

If the measured or calculated value exceeds the reference level, it does not necessarily follow that the basic restriction will be exceeded. However, whenever a reference level is exceeded it is necessary to test compliance with the relevant basic restriction and to determine whether additional protective measures are necessary. Compliance with the present guidelines may not implicitly involve interferences with, or effects on, medical devices such as metallic prostheses, cardiac pacemakers and defibrillators, and cochlear implants [71]. Interference with pacemakers may occur at levels below the recommended reference levels.

The exposure to electromagnetic fields at the frequencies above about 100 kHz normally results in significant energy absorption and a temperature increase in the body. In general, exposure to electromagnetic field in far-field conditions results in a highly nonuniform deposition and distribution of energy within the body, which must be assessed by dosimetric measurement and calculation. In the case of the frequencies in the range from about 20 MHz to 300 MHz, relatively high absorption can occur in the whole body, and to even higher values if partial body (e.g., head) resonances are considered.

As it was indicated in (1), in tissues, SAR is proportional to the square of the internal electric field strength and depends on the following factors:

- the parameter of the incident field, such us the frequency, intensity, polarization, and source configuration (near- or far-field);
- the characteristics of the exposed body, its size and internal and external geometry and the dielectric properties of the various tissues;
- ground effects and reflector effects of other objects in the field near the exposed body.

In the case of the near field conditions, it has been demonstrated that the near-field exposure can result in high local SAR (e.g., in the head, wrists, and ankles) and that whole-body and local SAR are strongly dependent on the separation distance between the high-frequency source and the body.

The basic restrictions for whole-body average SAR and localized SAR for frequencies between 10 MHz and 10 GHz, provided by ICNIRP [8], are presented in Table 2.

The biological and health effects in the frequency range from 10 MHz to a few GHz consist of a body temperature rise of more than 1°C. This level of temperature increase is due to exposure of individuals under moderate environmental conditions to a whole-body SAR of approximately 4 W kg^−1^ for about 30 min. As it is shown in Table 2, a whole-body average SAR of 0.4 W kg^−1^ has been chosen as the restriction that provides adequate protection for occupational exposure. A more restrictive limit is provided for exposure of the public: an additional safety factor of 5 is introduced resulting an average whole-body SAR limit of 0.08 W kg^−1^.

The frequency of 2450 MHz microwave ovens belongs to the range of 10 MHz to 10 GHz that is shown in Table 2. The more restrictive level of SAR is the whole-body average of 0.08 W kg^−1^ for the general public. After analyzing Figure 15, it follows that at the distance of 1 meter (far field conditions), the SAR reaches the value of 8 W kg^−1^ in localized areas above the legs; this value is between the levels for occupational and general public of localized SAR. Therefore, the basic restrictions of the SAR values for the general public are exceeded, although the obtained values are under the levels for occupational exposure.

Taking into account that the microwave ovens on the consumer market operate in 2.45 GHz ISM band, in the United States, the FCC regulates the ovens under 47 CFR Part 18, which applies to ISM equipment but specifically excludes communications equipment. While the FCC does not limit in-band emissions from ISM devices, manufacturers must nevertheless comply with FCC limits for human exposure to RF energy. In addition, the US Food and Drug Administration (FDA) limits the power density from leakage from a microwave oven to 50 W m^−2^ at a distance of 5 cm from the oven. Several studies have reported about the levels of exposure due to the leakage from ovens, and all agree that the measured values are far below the FDA limit [72].

The reference levels of the power density provided by ICNIRP [8], for the frequency range of 2–300 GHz, are presented in Table 3.

The restriction provided by the ICNIRP about the average whole body leakage irradiation exposure may not exceed a power density of 10 W m^−2^ (1 mW cm^−^²), taking into account the most restrictive limit. This value corresponds with an electric field of 61.4 V/m, which is considerably higher than the obtained values in this study.

## 6. Conclusions

In this work, the leaked radiofrequency power from an operating domestic microwave oven and the exposure levels it generates have been estimated. By using a hybrid simulation approach, combining full wave simulation coupled with in-house developed 3D ray launching code, estimation of the received* E*-field can be obtained for the complete volume of a complex indoor scenario. An equivalent model based on the application of a uniform* E*-field source distribution on the overall surface of the microwave oven has been employed, in order to provide equivalent antennas, which can be effectively introduced within the deterministic simulation code, hence reducing the overall simulation time. The microwave oven leakage is time and frequency dependent, which requires performing a multiple frequency analysis in order to characterize potential exposure as a function of operating time of the microwave oven heating process. Simulation results have been compared with spectral measurements as well as with personal dosimeter values, showing good agreement. The results confirm that exposure levels are strongly dependent on the location as well as on the topology and morphology of the indoor scenario, and although the microwave oven's leakage can degrade the quality of a wireless communication, as can be seen in the bibliography, the obtained electric field values are well below the limit values specified by ICNIRP. Diversity in the models of microwave ovens has been taken into account as a function of maximum output power, in the range of 750 W to 1800 W, obtaining estimations of leakage field exposure within this range.

Scenario complexity has also been taken into account by introducing an in-house developed simplified human body model, which can also be coupled to the deterministic 3D ray launching code. By considering the introduction of the human body model, modification in overall* E*-field levels can be assessed, as well as initial estimations of SAR values, which can be employed in order to analyze compliance with different regulations. Moreover, the obtained values are compared with these regulations, showing compliance for the complete scenario under analysis in the case of using a conventional microwave oven operating with a maximum output power of 800 W.

This technique allows performing assessment of exposure levels in complete scenarios, with feasible computational cost, aiding in the adoption of exposure reduction measurements, such as the identification of the optimal location of potential radiofrequency emitting sources, such as microwave ovens. Clear topological dependence, as well as the influence of maximum output power and the inclusion of human body models, indicates that estimations based on deterministic channel modelling offer valuable insight in order to assess potential impact of sources such as microwave ovens, especially in indoor environments. This analysis in the future can be extended to other types of devices, such as household appliances or industrial equipment.

## Figures and Tables

**Table 1:** Exposure limits for RF energy at the frequency band of 2.4 GHz.

ICNIRP general public	ICNIRP occupational	IEEE 95.1 controlled environments	IEEE 95.1 controlled environments	SanPiN occupational		SanPiN public
				*t* > 8 h	*T* < 0.2 h/day	
61.4 V/m	137 V/m	173.66 V/m	77.66 V/m	9.7 V/m	61.4 V/m	3.14 V/m

**Table 2:** Basic restrictions of the SAR for frequencies from 10 MHz to 10 GHz.

Exposure characteristics	Whole-body average SAR (W kg^−1^)	Localized SAR (head and trunk) (W kg^−1^)	Localized SAR (limbs) (W kg^−1^)

Occupational exposure	0.4	10	20
General public exposure	0.08	2	5

**Table 3:** Reference levels of power density from 2 MHz to 300 GHz.

Exposure characteristics	Power density (W m^−2^)

Occupational exposure	50
General public exposure	10
